# The Cure of Hernia

**Published:** 1903-08-29

**Authors:** 


### THE CURE OF HERNIA.

In an address delivered recently before the South-West London Medical Society, at the Bolingbroke Hospital, Sir Victor Horsley discussed the present methods of dealing with inguinal and femoral herniæ. The troubles which a rupture gave rise to, he said, might be classed under the two heads of discomfort and danger. The former of these was amply sufficient to justify operative methods. He contended that to apply a truss was not to treat the hernia, as it was only in the case of congenital hernia that a cure would be effected by this means. On the other hand a surgical operation offered such a good chance of permanent relief that it was called for in nearly every case; that, in fact, it was the only efficient and correct treatment unless some special contra-indication was present such as old age or debility. The dangers of surgical interference consisted of the risks of the anæsthesia and the risks of sepsis. Both of these he considered to be negligible in practice, and he thought it was justifiable in the case of a nervous subject to say that there was no danger at all. No fatality had occurred among the large number of such operations performed by his colleagues and himself during the last five years at University College Hospital. Sir Victor Horsley referred to the great difficulties that were in the way of ascertaining the ultimate results of surgical operations, and he thought it would be an excellent thing if an association were started with the object of finding out and recording after results. He was of opinion himself that there would be no recurrence of hernia if the operation were performed aright, but that faulty technique was responsible for a recrudescence of the trouble when such did occur. In inguinal hernia the operation which was most likely to lead to a permanent cure included a displacement of the lowest portion of the obliquus internus so as to fill up the weak spot in the abdominal wall with a layer of muscular tissue, as this was not liable to stretch and give way under pressure from the abdominal viscera. If there were an undescended testicle associated with the hernia, Sir Victor Horsley did not think it was right treatment to remove the testicle. He had yet to meet the case where it was impossible to place the testicle in the scrotum. With regard to femoral hernia he pointed out that that the femoral canal in an adult is from 1 to 1¼ inches long, and in a short but very interesting historical review of the various operations which have been from time to time devised for the cure of the rupture he demonstrated that the earliest procedures were limited to closing the lower end of the canal and that subsequently there had been a constant tendency with each new operation invented to attack a still higher portion of the canal with a view to its occlusion, but so far no procedure had been introduced which satisfactorily closed the very mouth of the canal. He went on to describe an operation which he had devised for satisfactorily attaining this end. The method was as follows:—An incision was made above and parallel to Poupart's ligament. The obliquus externus was divided along this incision, and then without further cutting, by retracting upwards the arching fibres of the transversalis and internal oblique the upper opening of the femoral canal was brought into view. The hernial sac was then emptied, its neck ligatured, and the sac was removed. The femoral canal was then occluded by the formation of a small osteo plastic flap taken from the posterior surface of the os pubis by means of a chisel. This flap was levered over the mouth of the femoral canal and fastened in place by a single suture. Sir Victor Horsley had performed this operation on three occasions, and had met with no unforeseen difficulties whatever during its performance. The results he thought would be superior to those obtained by other methods because it occluded the orifice of the femoral canal more satisfactorily, while it ran no risk of pressing upon or otherwise damaging the femoral vein.

